# Case report: Targeting the PD-1 receptor and genetic mutations validated in primary histiocytic sarcoma with hemophagocytic lymphohistiocytosis

**DOI:** 10.3389/fimmu.2023.1127599

**Published:** 2023-03-08

**Authors:** Yan Zhao, Yating Deng, Yi Jiang, Wenli Zheng, Yanlin Tan, Zhiwu Yang, Zhihua Wang, Feng Xu, Zhao Cheng, Lingli Yuan, Hongling Peng

**Affiliations:** ^1^ Department of Hematology, The Second Xiangya Hospital, Central South University, Changsha, Hunan, China; ^2^ Department of Pathology, The Second Xiangya Hospital, Central South University, Changsha, Hunan, China; ^3^ Department of Imaging, The Second Xiangya Hospital, Central South University, Changsha, Hunan, China; ^4^ Department of Hematology, Yiyang Central Hospital, Yiyang, Hunan, China; ^5^ Department of Thyroid and Breast Surgery, The Second Xiangya Hospital, Central South University, Changsha, Hunan, China; ^6^ Hunan Engineering Research Center of Cell Immunotherapy for Hematopoietic Malignancies, Changsha, Hunan, China

**Keywords:** histiocytic sarcoma, hemophagocytic lymphohistiocytosis, sequencing, programmed death-ligand 1 (PD-L1), targeted therapy

## Abstract

Histiocytic sarcoma (HS) is a rare hematological malignancy with limited treatment options, and it is also prone to complications such as hemophagocytic lymphohistiocytosis (HLH) in the later stages of the disease, leading to difficulties in treatment and poor prognosis. It highlights the importance of developing novel therapeutic agents. Herein, we present a case of a 45-year-old male patient who was diagnosed with PD-L1-positive HS with HLH. The patient was admitted to our hospital with recurrent high fever, multiple skin rashes with pruritus throughout the body and enlarged lymph nodes. Subsequently, pathological biopsy of the lymph nodes revealed high expression of CD163, CD68, S100, Lys and CD34 in the tumor cells and no expression of CD1a and CD207, confirming this rare clinical diagnosis. Concerning the low remission rate by conventional treatment in this disease, the patient was administered with sintilimab (an anti-programmed cell death 1 [anti-PD-1] monoclonal antibody) at 200 mg/d combined with a first-line chemotherapy regimen for one cycle. Further exploration of pathological biopsy by using next-generation gene sequencing led to the use of targeted therapy of chidamide. After one cycle of combination therapy (chidamide+sintilimab, abbreviated as CS), the patient achieved a favorable response. The patient showed remarkable improvement in the general symptoms and laboratory examination results (e.g., elevated indicators of inflammation); even the clinical benefits was not persistent, he survived one more month after his cessation of treatment by himself due to economic difficulty. Our case suggests that PD-1 inhibitor coupled with targeted therapy might constitute a potential therapeutic option for primary HS with HLH.

## 1 Introduction

Hemophagocytic lymphohistiocytosis (HLH) is a life-threatening hyperinflammatory syndrome caused by an abnormal activation of cytotoxic T lymphocytes (CTL) and natural killer (NK) cells. Clinically, the symptoms of HLH are non-specific, with an acute or subacute presentation (1) that primarily include persistent fever, lymphadenopathy, hepatosplenomegaly, complete hemocytopenia, and elevations in characteristic inflammatory biomarkers such as serum ferritin and soluble IL-2 receptor-α (sIL-2Rα) (2). HLH comprises two distinct forms, including a primary autosomal recessive form, also referred to as familial hemophagocytic lymphohistiocytosis (FHL), and a secondary HLH (sHLH) (3). sHLH is frequently triggered by infection or malignancy, and also by autoinflammatory/autoimmune diseases. Despite that 40% of HLH cases occur in adults, there are few scientific analyses, treatment trials, or clinical guidelines regarding HLH for adults (4, 5). If not treated promptly, primary HLH is usually fatal, and the mortality rate for sHLH or HLH in adults is also quite high (6). Therefore, it is of paramount importance to improve the prognosis and prolong the survival time of HLH patients.

Cases related to sHLH caused by hematologic neoplasms have been a hot topic in recent decades. Histiocytic sarcoma (HS), a subset of non–Langerhans’-cell histiocytosis, is a rare, aggressive, and poorly understood disease. HS occurs principally in adulthood, and is more prevalent in males. The clinical manifestations of HS comprise the entire spectrum from localized isolated masses in individuals to severe disseminated disorders, typically accompanied by lymph node and extra-nodal lesions; and encompassing the skin, connective tissue, the gastrointestinal tract, and the hematopoietic system (7). The diagnosis of HS is currently chiefly based on cytomorphology and immunohistochemical markers such as CD68, CD163, CD4, and lysozyme (8). Localized HS can generally be cured with a combination of surgical resection, chemotherapy, and radiotherapy. However, effective treatment for the disseminated disease has rarely been reported due to its rare and aggressive nature (9). Fortunately, with recent intensive studies on the molecular characterization of histiocytic neoplasms (HN), the utilization of targeted small molecule inhibitors has resulted in remarkable achievements in the treatment of HN, even obtaining continued disease-free survival of over two years (10–12). For example, antibodies against programmed cell death protein 1 (PD-1)/its ligand (PD-L1) (small molecule inhibitors that have been newly authorized in China [e.g., sintilimab]) have been proven to be effective in the treatment of various tumors, such as HN (13) and Hodgkin’s lymphoma (14). However, it still remains to be determined concerning their efficacy in rare diseases such as HS with HLH.

Herein, we presented a rare case of PD-L1-positive HS with HLH, and demonstrated a promising effect of PD-1 inhibitor in combination with chidamide on this aggressive disease. It is expected that our collective results will provide a novel perspective on the treatment of refractory HS.

## 2 Case report

A 45-year-old man (body weight of 47.5 kg, height of 158 cm and body surface area of 1.46 m^2^) was initially admitted to our hospital on July 31, 2021 due to recurrent fever with a maximal body temperature of 40°C for over two months and generalized rashes with uneven surface and pruritus. The patient had previously been diagnosed with a “drug rash” at the local hospital and was given anti-allergy therapy, yet without amelioration. One week previously, the patient developed an obvious shortness of breath, accompanied by a more frequent outbreaks of rash.

**Figure 1:**
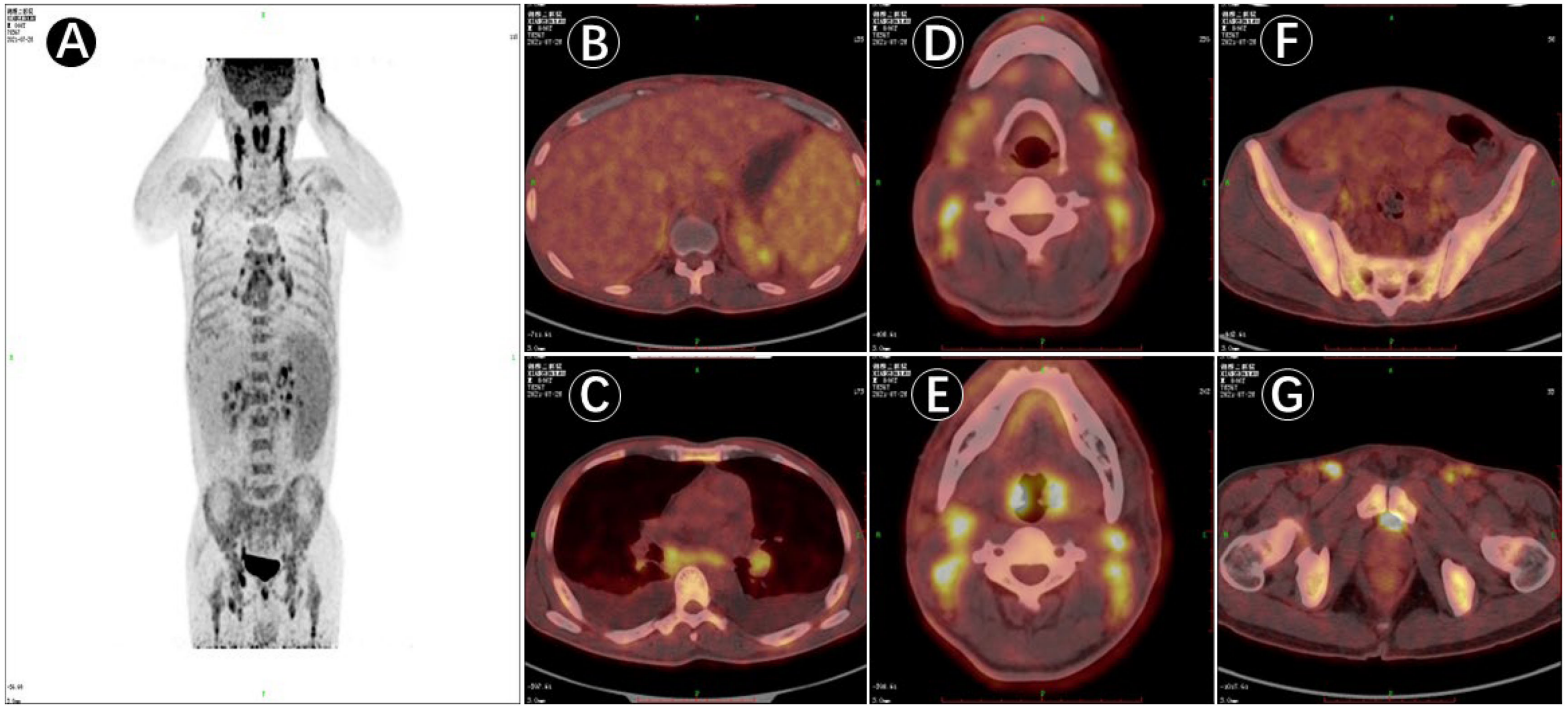
PET/CT image of ^18^F-deoxyglucose (FDG) uptake in the patient (male, 45 years of age) with multiple lymph node enlargement. **(A)**. PET/CT maximum intensity projection (MIP) image displaying multiple groups of metabolically elevated lymph nodes throughout the body, with bilaterally general symmetry and mildly increased splenic metabolism. **(B–G)**. Cross-sectional images in PET/CT showing generalized multiple enlarged lymph nodes, hepatosplenomegaly, and bilateral abnormally enlarged tonsils together with increased metabolism; and active involvement of generalized bone metabolism.

Upon admission, the patient was observed with clinical manifestations of pale complexion, poor spirit, cachexia, hepatosplenomegaly, dark erythema, and papules of varying sizes on both palms. Multiple enlarged lymph nodes could be palpated on both sides of his neck, supraclavicular and inguinal regions; with the largest node of 2.0 cm × 2.0 cm in size on the right side of his neck. Initial laboratory data on July 31, 2021, showed complete hemocytopenia, worsening inflammatory indicators, and elevated lactate dehydrogenase (LDH). In addition, serum ferritin was increased to 1519.25ng/mL (reference range, 21.80–274.66 ng/mL) on 21 August (**Table 1**). Subsequent hybrid imaging with 2-deoxy-2-[^18^F]fluoro-D-glucose positron emission tomography/computed tomography (2-[^18^F]FDG PET/CT) revealed generalized and multiple enlargements of the lymph nodes, hepatosplenomegaly, and bilateral tonsillomegaly—with augmented glucose metabolism throughout the body (**Figure 1**). The patient was therefore first considered to have lymphoma possibly, with a requisite for lymph node biopsy.

**Table 1:** Partial biochemical indicators from July to October 2021^*^.

Time	Complete blood count					Inflammatory indicators				Other biochemical indicators				
	WBC (3.5–9.5×10^9^/L)	RBC (3.8-5.1×10^12^/L)	PLT (125–350×10^9^/L)	Hb (115–150 g/L)	NEUT (1.8–6.3×10^9^/L)	PCT (0–0.05 ng/mL)	CRP (0.00–6.00 mg/L)	ESR (0–15 mm/h)	IL-6 (0–7 pg/mL)	LDH (120–250 μ/L)	sIL-2R (223–710 U/mL)	Ferritin (21.80–274.66 ng/mL)	ALB (40–55 g/L)
2021/7/31	1.69	1.96	29	55	0.75	21.00	76.30	103	34.20	327.0	–	–	25.1
2021/8/13	4.89	1.89	22	55	2.71	0.682	72.60	>140	9.43	–	–	1282.57	34.9
2021/8/21	5.82	2.05	14	60	5.10	0.303	47.50	>140	–	–	–	1519.25	35.5
2021/8/27	7.82	1.47	19	44	4.53	0.424	43.00	>140	–	283.0	11340	–	32.7
2021/9/15	2.26	1.61	21	48	1.36	0.380	31.83	>140	–	–	–	–	29.1
2021/9/17	2.54	1.92	26	58	1.19	0.575	38.12	53	–	335.7	–	1196.83	30.2
2021/9/29	2.22	2.10	21	62	1.31	69.00	112.11	59	111.00	536.9	–	10692.38	30.8
2021/10/4	1.69	1.77	11	54	1.15	1.120	12.10	–	–	–	–	–	30.4
2021/10/10	1.09	1.72	20	53	0.75	0.212	–	–	–	140.0	–	1686.97	29.9

^*^Reference ranges for each parameter are indicated in parentheses.

Supportive therapy was promptly started with anti-infective and anti-allergic medications, etc. However, the patient had no improvement in hematologic parameters (**Table 1**), and in fact showed deterioration (Hb of 44 g/L, PLT count of 19 × 10^9^/L, and β2-microglobulin [BMG] of 4.47 mg/L compared with normal prior value). CRP and PCT were also assessed for severe acute inflammation and were found to be elevated. Lymph node aspiration, bone marrow (BM) cytology, and biopsy were then performed to exclude the possibility of malignancies.

To investigate the potential complication of this patient, we further examined the soluble interleukin-2 receptor sIL-2R/sCD25 (ELISA instructions were followed: the anti-sIL-2R antibody was reacted with the serum as tested and the final A450 value was measured using an enzyme marker to analyze sIL-2R expression. [Kindstar Diagnostics, Wuhan, Hubei, China]) and NK cell activity (Peripheral blood was drawn on an empty stomach and NK cell activity (CD_16+56_^+^) is detected using a test kit and flow cytometry.). Laboratory results showed an increase in sIL-2R to 11,340 U/mL, and NK cell activity of 3.0%, indicating diminished NK cell activity. A diagnosis of HLH was confirmed combined with the patient’s medical history (5) (**Table 2**). Hence, the patient was provided with a revised HLH-1994 regimen (etoposide at 100 mg qw and dexamethasone at 10 mg/m^2^ qd). However, a joint consultation regarding the sampled pathologic specimen (i.e., tissues collected by aspiration from the right inguinal lymph node) showed predominantly follicular dendritic cells (FDC) hyperplasia, with insignificant cellular anisotropy and fair proliferative activity, making it difficult to diagnose malignancy at that time; and the result of BM biopsy was consistent with chronic inflammation, with no definite evidence of neoplasm. Owing to the undetermined pathologic diagnosis and inconspicuous treatment efficacy, the treatment regimen of HLH-1994 was suspended on September 2.

**Table 2:** Rationale for the diagnosis of HLH.

Diagnostic criteria for HLH based upon the HLH-2004 trial	
A diagnosis of HLH was established when any of the following two criteria were fulfilled:	
(1) a molecular diagnosis consistent with HLH—i.e., pathological mutations in *PRF1, UNC13D, STX11, STXBP2, Rab27a, LYST, SH2D1A, BIRC4, ITK, AP3β1, MAGT1*, and *CD27*;	
OR (2) when five of the eight indicators listed below were met:	This patient (YES/NO)
(i) fever: body temperature >38.5°C for >7 d;	YES
(ii) splenomegaly;	YES
(iii) hemocytopenia (involving both or all three peripheral blood lines): hemoglobin <90 g/L, platelets <100 × 10^9^/L, and neutrophils <1.0 × 10^9^/L; and not due to reduced bone marrow hematopoiesis.	YES
(iv) hypertriglyceridemia and/or hypofibrinogenemia: triglycerides >3 mmol/L and fibrinogen <1.5 g/L;	NO
(v) phagocytosis found in the bone marrow, spleen, liver, or lymph nodes;	NO
(vi) ferritin ≥ 500 μg/L;	YES
(vii) reduced or absent NK-cell activity; and	YES
(viii) elevated sCD25 (soluble interleukin-2 receptor).	YES

**Figure 2:**
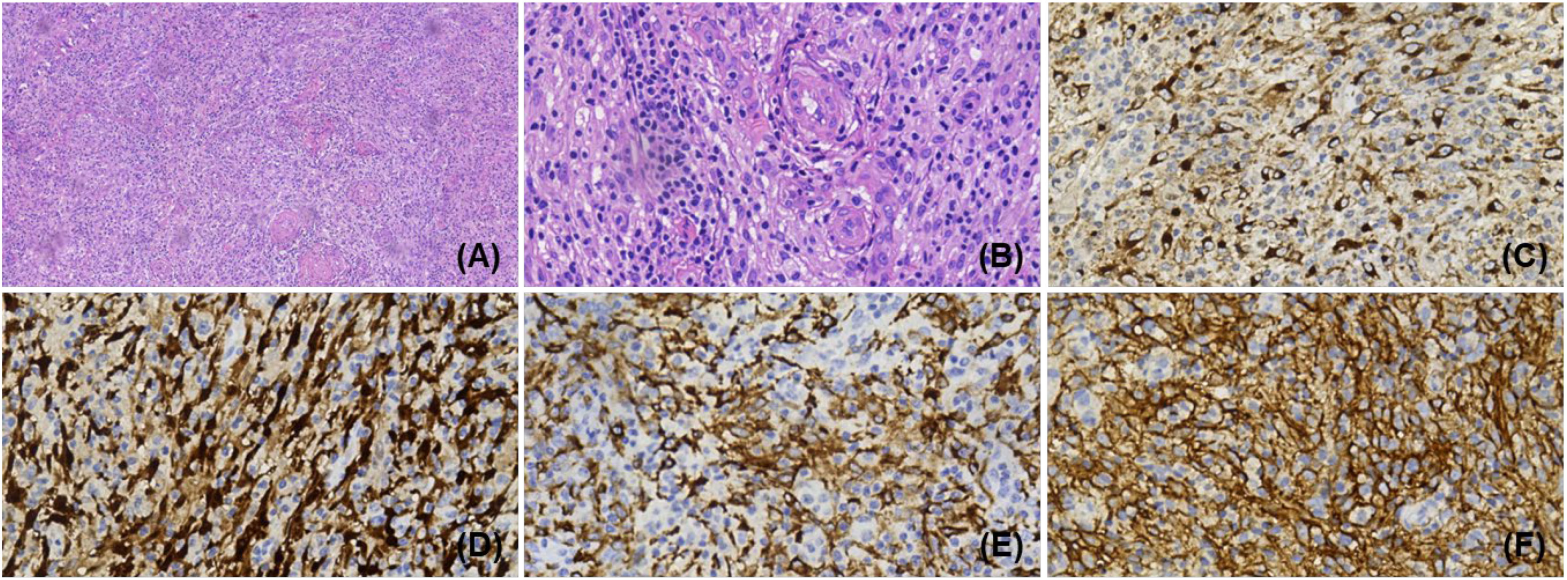
Pathologic histology of histiocytic sarcoma. **(A)**, Structural disruption of the lymph nodes under the microscope, along with marked proliferation of small vessels and a diffuse distribution of cells with slightly larger nuclei and abundant cytoplasm (H&E staining, ×100). **(B)**, Severe inflammatory cell infiltration in the alternating bright and dark areas; distribution of lymphocytes and plasma cells around the vessels in a target ring-like pattern (H&E staining, ×400). Immunostaining of tumor cells with positive staining for **(C)**, CD68 and **(D)**, S-100, respectively (IHC, × 400). **(E)**, Membrane staining for the most specific histiocytic marker, CD163^+^ (IHC, × 400). **(F)**, Diffuse staining for PD-L1^+^ in the tumor cells (IHC, × 400).

Biopsy of the right supraclavicular lymph node was ultimately performed on September 3 after a general surgery consultation, and the final pathologic diagnosis was consistent with primary HS (**Figure 2**). The sections for hematoxylin-eosin (H&E) staining (**Figures 2A, B**) revealed structural disruption of the lymph nodes with patches of large neoplastic cells that were markedly atypical cytologically, proliferation of small vessels, and diffusely distributed cells with slightly larger nuclei and abundant cytoplasm. Extensive immunohistochemical studies (**Figures 2C–E**) showed that the tumor cells were positive for CD163, CD68, S100, Lys, and CD34; and negative for CD1a, CD207, HMB45, MPO, and CD30. The Ki-67 proliferation index was 30%. Further immunostaining for PD-L1 was positive as over 80% of the tumor cell membranes were stained (**Figure 2F**). The patient unfortunately had a recurrent high fever on September 8 during supportive therapy, with generalized and scattered punctate rashes that were bright red in color and faded upon pressure; and the patient also exhibited splenomegaly that was assessed as progressive disease (PD). These attributes suggested highly malignant behaviors of this tumor.

**Figure 3:**
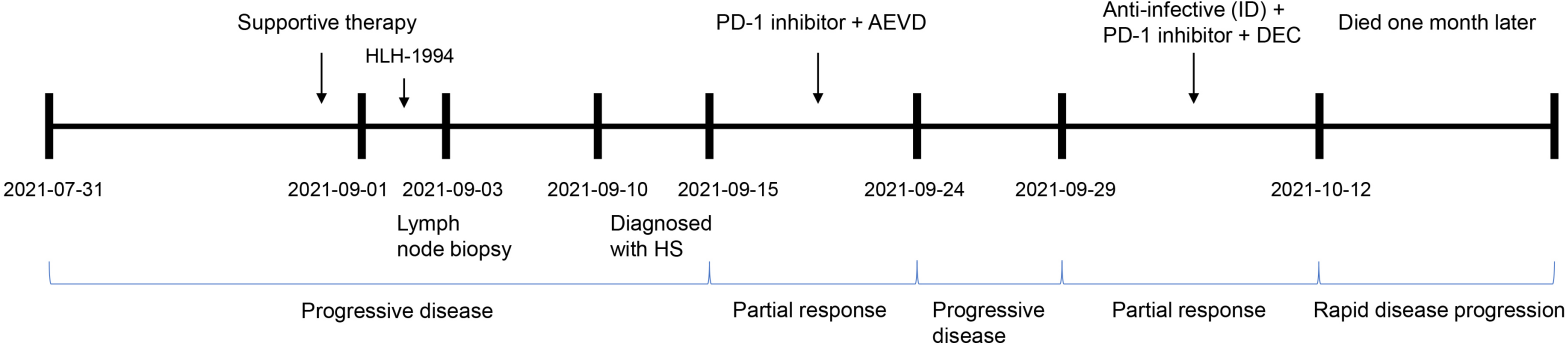
The clinical process comprising the patient’s treatment. Supportive therapy: anti-infective therapy, anti-allergic therapy, dexamethasone to reduce fever, and blood transfusion. PD-1 inhibitor, sintilimab; AEVD, epirubicin (50 mg on d 1), etoposide (100 mg on d 1), vincristine (3 mg on d 1) and dexamethasone (15 mg on d 1); ID, intravenous drip; DEC, dexamethasone (10 mg on d 0–14), etoposide (100 mg on d 1–2 and d 8) and chidamide (20 mg biw; i.e., every Wednesday and Saturday); PD, progressive disease; and PR, partial response.

There is presently no uniform standard therapeutic regimen for HS. Importantly, PD-L1 was also highly expressed in this patient. A combination chemotherapeutic regimen of PD-1 inhibitor + AEVD (epirubicin/etoposide/vincristine/dexamethasone) was implemented on September 15 (**Figure 3**) that consisted primarily of sintilimab (200 mg on d 0), epirubicin (50 mg on d 1), etoposide (100 mg on d 1), vincristine (3 mg on d 1), and dexamethasone (15 mg on d 1) in a 21-d course—supplemented by supportive treatment. The patient was fortunately found to improve after 2 days of the combination chemotherapy. On September 17, 2021, the patient was observed with an improvement in the general symptoms, showing alleviation in generalized rashes, and no fever, coughing, or expectoration. Multiple lymph nodes shrank and the levels of inflammatory indicators also decreased. In addition, Hb was improved to 58 g/L, PLT to 26×10^9^/L, PCT to 0.575 ng/mL, CRP to 38.12 mg/L, and ferritin to 1196.83 ng/mL (**Table 1**). The patient was discharged on September 18.

However, one week after discharge, the patient developed a rapid growth of the lymph nodes after a reduction in size previously. The largest one was now 3.0 cm × 3.0 cm in size on the right side of the neck, and was tough and poorly mobile. The patient also had persistent fever (37–40.5°C). The patient was admitted to our hospital for the second time on September 29, 2021 for chemotherapy. Physical examination of the patient after admission showed recurrent rashes. Emergency hematologic evaluation revealed triglyceride level of 3.07 mmol/L, BMG of 4.40 mg/L, IL-6 of 111.00 pg/mL, PCT of 69.00 ng/mL, whole blood CRP of 112.11 mg/L, LDH of 536.9 μ/L, and a significantly elevated ferritin of 10,692.38 ng/mL; accompanied by a dramatic deterioration in the condition of the patient (**Table 1**). The patient exhibited an intermittent fever (T_max_ of 40.5°C), coughing and expectoration. The patient was provided with antibiotic treatment on admission, and a second chemotherapy was initiated on September 30.

**Figure 4:**
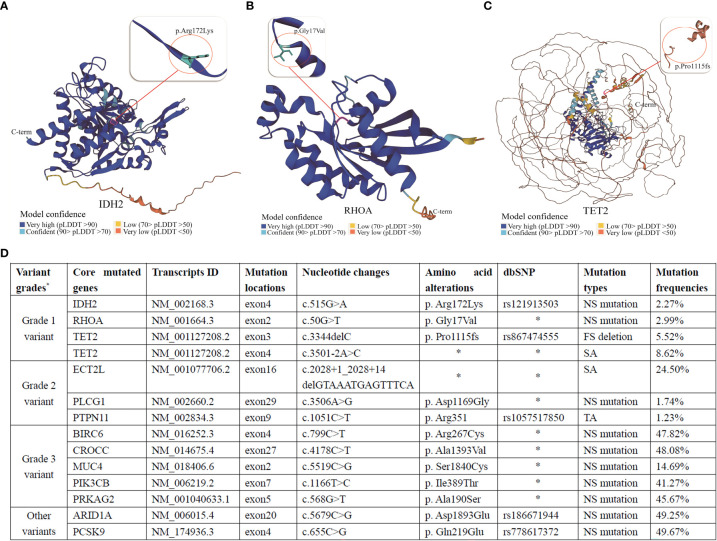
Gene mutations detected in the patient: **(A)**. IDH2, **(B)**. RHOA, and **(C)**. TET2. **(D)**. Specific information on the primary mutated genes provided by the sequencing company. NS mutation, non-synonymous mutation; FS deletion, frame-shift deletion; SA, spliceosome abnormalities; TA, terminator acquisition.

In order to seek for better treatment choices, next-generation gene sequencing (NGS) of the pathological biopsy samples from our patient was ordered subsequently. Using NGS technology, the first puncture biopsy of cervical lymph nodes detected mutations in *IDH2*, *RHOA*, *TET2*, *ECT2L*, *PLCG1*, *PTPN11*, *BIRC6*, *CROCC*, *MUC4*, *PIK3CB*, *PRKAG2*, *ARID1A*, and *PCSK9* genes—with primary variants of *IDH2*, *RHOA*, and *TET2* (**Figure 4**). A novel missense mutation [c.50G>T, p.Gly17Val (NM_001664)] was also found in *RHOA* gene; non-synonymous mutation [c.515G>A, p.Arg172Lys (NM_002168)] in *IDH2* gene; and c.3344delC, p. Pro1115fs (NM_001127208) in *TET2* gene, with a frame-shift deletion mutation and a splicing mutation at c.3501-2A>C (NM_001127208). These mutations are known to be damaging mutations associated with high response to drugs classified in the epigenetic modification category, i.e., histone deacetylase inhibitors (HDACis) (15).

The HDACis chidamide was consequently administered in this case and the specific regimen of chemotherapy was changed to sintilimab at 200 mg on d 0, dexamethasone at 10 mg on d 0–14, etoposide at 100 mg on d 1–2 and d 8, and chidamide at 20 mg (biw, every Wednesday and Saturday). After 4 days of treatment, review of the patient indicated a marked remission of inflammatory indicators, and after 10 days of targeted therapy, PCT was recovered to 0.212 ng/mL, LDH to 100 μ/L, and ferritin to 1686.97 ng/mL (**Table 1**). Moreover, the fever disappeared and the infection was controlled in the patient. The enlarged lymph nodes were also markedly diminished to the size of a soybean. The patient was discharged with instruction of chidamide at 20 mg biw and was asked to be back for next cycle in one week.

However, the patient discontinued chemotherapy due to financial problem and survived for another month.

## 3 Discussion

Primary HS is a rare hematopoietic malignancy that can occur alone or in conjunction with other hematologic tumors such as non-Hodgkin’s lymphoma, myelodysplasia, or acute leukemia. HS with HLH is, in fact, a relatively rare disease clinically, with only eight cases in total reported in the literature (16–23). A review of multiple patient cohorts suggests an average age at presentation of approximately 63 years old (24). Importantly, the clinical prognosis of HS with HLH is extremely poor in these patients. HS has no specific clinical manifestations, and patients usually present with fever, night sweats, or weight loss; and its diagnosis depends largely on the pathological findings.

Considering its rarity and histological overlap with various clinical phenotypes, the diagnosis of HS can be extremely challenging. The differential diagnosis of HS involves various lymphomas, other histiocytic and dendritic cell tumors, melanomas, pleomorphic sarcomas and angioimmunoblastic T-cell lymphoma (AITL) (25). AITL is the classic form of mature T-cell lymphoma of T-follicular helper (TFH) origin. Specifically, apart from a clinical phenotype resembling HS, AITL has been identified with specific genetic findings, including recurrent somatic mutations in *TET2*, *RHOA*, *IDH2*, *CD28*, and *DNMT3A*, as well as *ITK-SYK* and *CD28-CTLA4* fusions (26). Histopathologically, the tumor cells in AITL possess different morphological manifestations and predominantly express T-cell antigens such as CD3, CD4, CD10, PD-1, CXCL13, BCL-6, CD40L and NFATC1 (27). Anaplastic large cell lymphoma (ALCL) is an important differential diagnosis to consider and is characterized by ‘hallmark’ cells with embryo-like nuclei and multiple prominent nucleoli (25). Anaplastic lymphoma kinase (ALK)-positive ALCL demonstrates rearrangements involving the ALK gene (e.g., t (2;5)) and overexpression of ALK protein. When tumor cells in ALCL exhibit histiocytic morphology, expression of CD30, epithelial membrane antigen (EMA), additional T-cell markers (other than CD4 and CD43) and deletion of CD163 and PU.1 distinguish T-cell lymphomas (e.g., ALCL and PTCL, NOS) from HS (25, 28). Most large B-cell lymphomas are positive for B-cell lineage markers that are not expressed in HS (25). For example, large tumor cells in classical Hodgkin’s lymphoma (CHL) show positivity for CD15 and CD30, weak positivity for PAX5, and negativity for CD45 and histiocyte markers (28). Diffuse large B-cell lymphoma (DLBCL) expresses B-cell markers (CD19, CD20, CD22, CD79a) and may also express germinal center markers (CD10, BCL6). In contrast, S100, CD1a, CD207/langerin were negative (28). Although both HS and myeloid sarcomas express histiocytic markers, the diagnosis of myeloid sarcoma can be differentiated by myeloid markers such as CD13, CD33 and myeloperoxidase (29). Melanomas are usually positive for Melan-A, HMB-45 and SOX10, but negative for CD163 (25). Further pathologic diagnosis is therefore necessary to clarify the type of specific diseases. In the era of individualized medicine, these findings may promote the trials of epigenetic modulators and/or immunotherapy.

The molecular landscape of HS is not well described at present. Previous studies have identified activating mutations of *PI3K/AKT/mTOR* and *RAS/MAPK* pathways in the majority of HS cases, which play an influential role in the pathogenesis of HS (30). Egan and colleagues (31) reported a high number of genetic alterations within the *RAS/MAPK* signaling pathway in 21 of 21 cases, with alterations in *NF1* (6/21), *MAP2K1* (5/21), and *PTPN11* (4/21). In addition, there were case reports that trametinib (a MEK1/2 inhibitor) and vemurafenib (a BRAF inhibitor) were effective in HS patients with mutations in the MAPK/ERK pathway (e.g., *BRAF ^V600E^*, *KRAS*, and *MAP2K1*) (32–35).

In the nucleus, HDAC mediates the deacetylation of various histones to modulate the silencing of downstream tumor suppressor genes and promote tumor development. By reversing this action, HDACis molecules can induce apoptosis and differentiation of tumor cells, designating a novel class of anti-tumor agents. For example, chidamide is a new HDACis that is currently used to treat relapsed and refractory peripheral T-cell lymphoma (R/R PTCL) (36). In our case, whole-exome sequencing on the pathologic tissue from cervical lymph nodes depicted mutations in *PTPN11*, *IDH2, RHOA*, and *TET2*, indicating the potential pathogenesis of HS. Similar to AITL (26, 36), the patient was given targeted therapy based on his genetic profile. For the first time, our study applied chidamide in the treatment of primary HS and demonstrated a partial response that is not persistent.

There is so far no standard therapeutic regimen for HS due to its rarity and invasiveness. Most patients reported in the literature received surgery, radiotherapy, and/or chemotherapy. For localized diseases, surgical resection combined with post-operative chemotherapy may be relatively effective if there is an opportunity for surgery at an early stage (37). However, further radiotherapy and combination chemotherapy are essential when the disease involves multiple foci (37). Temozolomide (TMZ); cyclophosphamide, doxorubicin, vincristine and prednisone (CHOP); ifosfamide, carboplatin and etoposide (ICE); and doxorubicin, bleomycin, vinblastine and dacarbazine (ABVD) are frequently used chemotherapeutics in clinical practice. There are also a few single-case reports showing favorable outcomes with allogeneic hematopoietic stem cell transplantation (allo-HSCT) (38). In one study, thalidomide treatment successfully halted disease progression in two HS patients after autologous HSCT (auto-HSCT) (39). However, these treatments are palliative in nature and possess poor therapeutic efficacy. Recently, tumor tissue NGS studies revealed that a number of mutations such as BRAF V600E and MAPK pathway (RAS-RAF-MEK-ERK) mutations have been described in HN(including non-LCH patients) (10, 11, 35). Furthermore, kinase fusions (e.g., *BRAF*, *ALK*, and *NTRK1*) may provide further novel therapeutic targets for patients with histiocytosis (11, 40). Therefore, apart from conventional treatments, the role of targeted therapies (BRAF and MEK inhibitors) is emerging accompanied by continued disease-free survival of over two years, based on small clinical trials and case reports (12, 40, 41). In a clinical phase II study, cobimetinib, a MEK1/2 inhibitor, was administered to adult patients with histiocytoses of any mutational status (12). The overall remission rate (ORR) was 89% (90% CI: 73-100) in 18 treated patients. At one year, remission was sustained in 100% of patients and 94% remained progression-free (12). Overall, these advances are promising but there is great heterogeneity in the patient population and these studies are limited to relatively small number of patients. In addition, checkpoint inhibitors and HDACi were not used in these studies compared to our case. Further studies remain to be conducted.

The use of immune checkpoint inhibitors (ICIs) (e.g., anti-PD-1/PD-L1 antibodies) brings survival benefits to cancer patients, which has recently developed into an exciting area in the field of cancer treatment research. PD-1 is a suppressor receptor that is primarily expressed on T cells. Under normal physiologic conditions, PD-1 suppresses T-cell activation and cytokine production by binding to its ligand (PD-L1) on tumor cells, thereby protecting the organism from autoimmune attack. ICIs can block the “tumor immune-escape mechanism” and restore the patient’s own immune system to fight against cancers. As previously reported, PD-L1 staining was positive on tumor cells in a minority of HS cases, providing a rationale for its use in immunotherapy of HS patients. For example, a recent report showed that PD-L1 was highly expressed in some HS patients, and that nivolumab, a PD-1 inhibitor, achieved acceptable therapeutic effect for HS patients (42). In some cases, even patients with HLH can be effectively treated by using ICIs (43). We herein selected a chemotherapeutic regimen of sintilimab combined with AEVD to address the high expression of PD-L1 in our patient and noted a definite improvement in the condition of the patient. It indicated that the PD-1 inhibitor may exert a certain therapeutic effect on HS patients who showed high PD-1 expression. While immunochemotherapy offers additional therapeutic opportunities for refractory HS, and monotherapy shows relatively low response rates in individuals.

To improve the efficacy of immunotherapy, Que et al. (44) further explored the synergistic anti-tumor efficacy of chidamide and PD-1 inhibitor, and demonstrated that chidamide stimulated PD-L1 expression in tumor cells *in vitro and in vivo*; while anti-tumor immunity was augmented by reinvigorated tumor-infiltrating CD8^+^ T cells. Similarly, Wei et al. (45) reported that chidamide recruited immune cells and enhanced the innate immune function of PD-1^+^ cells (e.g., CD8^+^/CD4^+^ T cells and NK cells) in PTCL, indicating that anti-PD-1 antibodies combined with chidamide constituted a potential novel approach to cancer treatment. In addition, other investigators also confirmed that their patient obtained a durable and complete molecular response after combined treatment using chidamide+sintilimab (CS), with only mild toxicity simultaneously (46). Mechanistically, HDACis can stimulate the expression of PD-L1 on tumor cells, which will strengthen the interaction with PD-1 on immune cells and reduce the immune response. It contributes to a synergistic anti-cancer effect when used in combination with ICIs. On the other hand, PD-1 inhibitors used alone may be prone to drug resistance, and HDACis is able to overcome this drawback.

Certainly, as mentioned above, some conclusions about the clinical application of ICIs and HDACi are currently based mostly on single cases and their therapeutic efficacy is subject to uncertainty (47, 48). Additional clinical trials and cohort studies of drugs in hematological tumors need to be conducted and validated. To our knowledge, our combination therapy with CS is the first-ever report on its use in HS patients with HLH. Among this, it remains to be considered whether the efficacy of treatment in the patient is related to the types of drugs, duration of treatment and drug doses, due to the short duration of the combination therapy. However, despite no observation of apparent toxic side effects with CS therapy, the patient had deteriorated condition again shortly after discharge from the hospital, manifested as persistent fever, as well as enlarged lymph nodes and spleen. Due to the aggressive nature of this disease and financial considerations, the patient discontinued chemotherapy and died one month later, which was ultimately considered as a PD-related death.

In summary, primary HS with HLH is a very aggressive disease with a low survival rate that portends the need for multiple modalities in combination therapy. Importantly, ICIs are effective in many diseases but are susceptible to drug resistance, possibly related to the down-regulation of tumor antigens or receptor levels (including PD-L1) and reduction in the number of tumor-infiltrating lymphocytes. We therefore optimized the NGS of the pathological biopsy tissue from the patient and uncovered *IDH2*, *RHOA*, and *TET2* mutations, suggesting the potential to use HDACi in targeted therapy. Based on the results of a phase I/II clinical study on the treatment of R/R-ENKTL by anti-PD-1 antibodies combined with chidamide (36), we conclude here that chidamide may overcome the drug resistance of ICIs, ameliorate the expression of PD-L1, and lessen the depletion of T cells by modulating the tissue microenvironment and other underlying molecular mechanisms, thereby achieving synergistic effects of the combined regimen. This patient reported in our study was given a combined therapeutic regimen of sintilimab with chidamide, etoposide, and dexamethasone for 21 days per course; and the patient showed improved symptoms to some extent after treatment. The combined treatment of chemotherapy and CS may be a promising therapeutic option in such patients with an otherwise dismal outcome. However, additional clinical trials are needed to validate the efficacy and underlying mechanisms of action of this therapeutic strategy.

## Data availability statement

The original contributions presented in the study are included in the article/supplementary material. Further inquiries can be directed to the corresponding authors.

## Ethics statement

The studies involving human participants were reviewed and approved by the ethics committee of the Second Xiangya Hospital of Central South University. The patients/participants provided their written informed consent to participate in this study. Written informed consent was obtained from the individual(s) for the publication of any potentially identifiable images or data included in this article.

## Author contributions

HP and LY were responsible for the study design and acquisition of data. YD for the acquisition and interpretation of data, and drafting of the manuscript. YJ, WZ, YT, ZY, and FX for the acquisition, analysis, and interpretation of the data and YZ for the acquisition, assembly, analysis and interpretation of the data, and the drafting of the manuscript and critical revisions to the manuscript. All authors contributed to the article and approved the submitted version.
